# Intra-articular adipose-derived cell therapies for knee osteoarthritis: a systematic review of randomized controlled trials

**DOI:** 10.3389/fmed.2026.1771008

**Published:** 2026-04-22

**Authors:** Joelington Dias Batista, Ludimila Dias Silva, Jobson Dias Batista, Gabrielly Santos Pereira, Marcelo Lourenço da Silva

**Affiliations:** 1 Orthopain Pain Institute, Anápolis, GO, Brazil; 2 Neuropsychobiology and Motor Behavior Laboratory, Faculty of Medicine of Ribeirão Preto, University of São Paulo – USP, Ribeirão Preto, Brazil; 3 Laboratory of Neuroscience, Neuromodulation and Study of Pain (LANNED), Federal University of Alfenas (UNIFAL-MG), Alfenas, MG, Brazil

**Keywords:** adipose-derived cell therapy, adipose-derived mesenchymal stromal cells, intra-articular injection, knee osteoarthritis, systematic review

## Abstract

**Introduction:**

Knee osteoarthritis is a leading cause of chronic pain, functional limitation, and disability worldwide, imposing a substantial socioeconomic burden. Despite advances in conservative management and intra-articular therapies, many patients experience limited or transient symptomatic relief, underscoring the need for biologically based interventions. Intra-articular adipose-derived cell therapies, including adipose-derived mesenchymal stem or stromal cells (ADSCs), stromal vascular fraction (SVF), and microfragmented adipose tissue (MFAT), have emerged as regenerative strategies aimed at modulating inflammation and joint homeostasis. This systematic review evaluated the efficacy, structural effects, and safety of intra-articular adipose-derived cell–based therapies for knee osteoarthritis in adults.

**Methods:**

Randomized controlled trials published between 2015 and 2025 were identified through systematic searches of PubMed, Embase, Scopus, and Web of Science. Eligible studies compared ADSCs, SVF, or MFAT with placebo, hyaluronic acid, platelet-rich plasma, corticosteroids, or conservative care, and reported outcomes on pain, function, imaging-based structural changes, and safety.

**Results:**

Nineteen randomized controlled trials met inclusion criteria. Across studies, adipose-derived interventions, particularly ADSC-based therapies, produced clinically meaningful reductions in pain and improvements in functional outcomes assessed by WOMAC, KOOS, and visual analog scales.

**Discussion:**

Several ADSC and SVF trials reported favorable magnetic resonance imaging findings, including improvements in cartilage quality, although consistent cartilage regeneration was not demonstrated. MFAT trials yielded heterogeneous results, often showing symptomatic benefits comparable to established injective therapies but limited structural effects. No serious treatment-related adverse events were reported. Intra-articular adipose-derived cell therapies are safe and provide meaningful pain relief and functional improvement in selected patients, with ADSCs showing the most consistent clinical signals.

**Systematic review registration:**

https://www.crd.york.ac.uk/PROSPERO/view/CRD420251241498, Identifier: CRD420251241498.

## 1 Introduction

Knee osteoarthritis (OA) is a highly prevalent degenerative joint disease associated with chronic pain, progressive functional limitation, and a substantial reduction in quality of life (1, 2). It is a multifactorial condition characterized by progressive articular cartilage degeneration, subchondral bone remodeling, low-grade synovitis, and biomechanical alterations that collectively contribute to disease progression (1, 3). In the context of population aging and rising obesity rates, knee OA represents a major public health challenge, exerting an increasing burden on healthcare systems and generating significant socioeconomic costs (4).

From a pathophysiological perspective, osteoarthritis is characterized by a progressive imbalance between catabolic and anabolic processes within the joint microenvironment (2). Chondrocytes exposed to mechanical overload and inflammatory stimuli exhibit increased expression of catabolic mediators, including matrix metalloproteinases (MMPs), aggrecanases (ADAMTS), pro-inflammatory cytokines such as interleukin-1β (IL-1β), interleukin-6 (IL-6), and tumor necrosis factor-*α* (TNF-*α*). These processes culminate in extracellular matrix degradation and progressive deterioration of joint function (5). Concurrently, subchondral bone alterations, osteophyte formation, and synovial activation sustain a chronic low-grade inflammatory state that is strongly associated with pain, stiffness, and functional impairment (6).

Currently available therapeutic strategies for knee OA are largely focused on symptomatic relief and functional improvement and include conservative measures, pharmacological therapies, and intra-articular interventions such as corticosteroids, hyaluronic acid (HA), and platelet-rich plasma (PRP). Clinical studies evaluating intra-articular HA injections have demonstrated pain reduction and functional improvement; however, these effects are often transient and dependent on formulation characteristics and patient profile (7). Systematic reviews of intra-articular interventions similarly report variable clinical responses to corticosteroids and PRP, with benefits frequently limited in duration and lacking consistent evidence of structural disease modification (8–10). Overall, these treatments have limited efficacy and generally fail to alter the structural course of the disease in a sustained manner. In advanced stages, total knee arthroplasty remains the definitive treatment option, although it is associated with high costs, surgical risks, and variable functional outcomes (11, 12).

In this context, biological therapies based on intra-articular administration of adipose-derived products have emerged as innovative strategies for the management of knee osteoarthritis. These approaches include adipose-derived stem/stromal cells (ADSCs or ADSCs), stromal vascular fraction (SVF), and microfragmented adipose tissue (MFAT). Although these products differ in terms of processing methods, cellular composition, and regulatory requirements, they share a common biological rationale. Adipose tissue represents an abundant and readily accessible source of cells with immunomodulatory, anti-inflammatory, and trophic properties capable of acting on multiple components of the joint microenvironment (13). Recent studies suggest that adipose-derived MSCs may offer promising clinical effects in pain reduction and functional improvement in knee OA, although methodological heterogeneity and mixed results warrant further investigation (14).

The therapeutic effects of adipose-derived cell therapies are thought to be mediated predominantly through paracrine mechanisms, involving the release of anti-inflammatory cytokines, growth factors, and extracellular vesicles. These mediators modulate the local immune response, reduce synovial activation, inhibit catabolic inflammatory pathways, and promote homeostasis of articular cartilage and subchondral bone (13, 15). Experimental and clinical evidence indicates that adipose-derived stromal cells act primarily through immunomodulatory and trophic signaling rather than direct differentiation into chondrocytes, interfering with key inflammatory pathways implicated in osteoarthritis, including IL-1β-, TNF-*α*-, and matrix metalloproteinase-mediated signaling (16, 17).

Accordingly, the physiological rationale of these interventions aligns closely with the central pathological mechanisms of osteoarthritis, suggesting potential for sustained symptomatic relief and, in selected contexts, disease-modifying effects, particularly in patients with early to moderate stages of knee OA (13, 14).

Preclinical evidence and early clinical studies have demonstrated pain reduction and functional improvement following adipose-derived cell-based therapies, as well as favorable structural findings on imaging in selected studies, including improvements in cartilage quality and quantitative magnetic resonance imaging parameters (18–20).

Nevertheless, the existing literature remains highly heterogeneous with respect to clinical indications, preparation techniques, biological dosing, number of injections, routes of administration, and comparator interventions. This heterogeneity limits cross-study comparability and the generalizability of findings (17, 21). Inconsistent results across trials, substantial methodological variability, and the coexistence of multiple distinct products under the umbrella term “adipose-derived cell therapy” complicate critical interpretation of the available evidence and its translation into routine clinical practice (13).

In light of these considerations, the objective of this systematic review was to synthesize and critically analyze randomized controlled trials evaluating intra-articular adipose-derived cell therapies for the treatment of knee osteoarthritis, with a focus on pain, functional outcomes, imaging-based structural changes, and safety. By integrating clinical, functional, and structural evidence, this review aims to provide a comprehensive and up-to-date assessment of the role of these therapies in knee OA management and to identify relevant knowledge gaps to guide future research.

## 2 Materials and methods

### 2.1 Study design

This systematic review synthesized randomized evidence on intra-articular adipose-derived cell therapies—including stromal vascular fraction (SVF), adipose-derived stem/stromal cells (ADSCs), and related adipose-derived products—for the management of knee osteoarthritis. All included interventions consisted exclusively of percutaneous intra-articular injections. Studies involving surgical implantation, scaffold-based delivery, or procedures requiring operative exposure were not considered eligible for inclusion in this review. The review focused on clinical outcomes (pain and function), structural outcomes (cartilage and imaging-based biomarkers), and safety (adverse events and complication rates). Eligible studies comprised randomized controlled trials and controlled clinical trials reporting short- and/or long-term follow-up. The review was conducted in accordance with PRISMA 2020 guidelines and was prospectively registered in PROSPERO (CRD420251241498).

### 2.2 Eligibility criteria

Eligibility criteria were defined according to the PICO framework.

Population: Adults (≥18 years) with clinical and/or radiographic diagnosis of knee osteoarthritis. Studies involving pediatric populations, preclinical or animal models, and mixed orthopedic populations in which knee osteoarthritis–specific data could not be extracted were excluded. Trials including cultured, genetically modified, or non–adipose-derived cell products were also excluded.

Intervention: Intra-articular administration of adipose-derived cell–based therapies, including expanded or cultured adipose-derived mesenchymal stem/stromal cells (ADSCs/AD-MSCs), stromal vascular fraction (SVF), and microfragmented adipose tissue (MFAT). It is important to note that SVF represents a heterogeneous cell population that includes ADSCs along with endothelial cells, pericytes, immune cells, and extracellular matrix components, rather than a purified ADSC product. In most clinical applications, SVF is administered in suspension with biological carriers such as plasma or platelet-rich plasma, which may influence its biological activity. These products were therefore analyzed as biologically distinct intervention categories.

*Comparison*: Eligible comparators included placebo or saline injection, hyaluronic acid, platelet-rich plasma, corticosteroid injections, standard conservative management, exercise-based rehabilitation, or other conventional intra-articular treatments without adipose-derived cell therapy.

*Primary outcomes*: Pain reduction assessed using validated instruments such as the Visual Analog Scale (VAS), Numeric Rating Scale (NRS), or pain-related subscales of WOMAC or KOOS.

*Secondary outcomes*: Functional outcomes including WOMAC total score, KOOS, IKDC, Lysholm, and other validated knee-specific measures; structural or cartilage-related outcomes evaluated by imaging modalities (MRI, CT, or radiography), such as cartilage thickness, volume, defect size, or semiquantitative scores (e.g., WORMS, MOAKS, MOCART); health-related quality of life; treatment failure or need for subsequent surgical intervention; and safety outcomes, including local or systemic adverse events, injection-related complications, and procedure-related morbidity.

*Study designs*: Eligible studies were randomized controlled trials (double-blind, single-blind, assessor-blind, or open-label). Case reports, observational cohort studies, uncontrolled trials, small case series, and conference abstracts without extractable data were excluded. Studies in which the independent effects of adipose-derived cell therapies could not be distinguished from concomitant surgical or pharmacological interventions were also excluded (Table 1).

**Table 1:** Study characteristics of the included studies.

Author	Study design	KOA severity (KL)	Sample size (completed)	Intervention	Comparator	Follow-up	Primary outcomes	Key findings
Lee et al. (18)	Phase IIb, double-blind RCT	KL II–III	24	IA autologous ADSCs (single injection)	Saline placebo	6 mo	WOMAC, VAS, MRI	Significant pain and functional improvement vs. placebo; MRI suggested cartilage defect improvement
Freitag et al. (24)	Single-blind RCT	KL II–III	30	IA autologous ADSCs	Hyaluronic acid	12 mo	WOMAC, VAS	ADSCs superior to HA for pain and function
Sajadi et al. (22)	Triple-blind RCT	KL II–III	60	IA allogeneic ADSCs	Saline placebo	12 mo	WOMAC, VAS	Significant improvements in pain and function vs. placebo
Richter et al. (31)	Double-blind RCT	KL II–III	118	IA microfragmented adipose tissue (MFAT)	Saline placebo	12 mo	VAS, KOOS	MFAT reduced pain and improved function vs. placebo
Pers et al. (17)	Phase IIb, double-blind RCT	KL I–III	146	IA autologous ex vivo–expanded ADSCs	Placebo	12 mo	WOMAC pain and function	ADSCs improved pain and function vs. placebo; favorable safety
Barfod et al. (33)	Double-blind RCT	KL II–III	120	IA MFAT	Saline placebo	24 mo	KOOS, VAS	MFAT not superior to placebo at 2 years
Zaffagnini et al. (34)	Prospective RCT	KL II–III	118	IA MFAT	PRP	24 mo	KOOS, IKDC	Comparable improvements between MFAT and PRP
Lu et al. (29)	Prospective RCT	KL II–III	80	IA autologous SVF	Conservative treatment	12 mo	WOMAC, VAS	SVF improved pain and function vs. control
Tangkanjanavelukul et al. (25)	Randomized, blinded-endpoint trial	KL I–II	60	IA autologous ADSCs	Hyaluronic acid	12 mo	WOMAC, MRI cartilage	ADSCs superior for symptoms and cartilage quality
Song et al. (19)	Randomized clinical trial	KL II–III	24	Repeated IA autologous ADSCs	Saline	24 mo	WOMAC, MRI	Sustained pain/function improvement; increased cartilage volume
Hong et al. (28)	Double-blind, self-controlled RCT (bilateral knees)	KL II–III	32 knees	IA SVF	Hyaluronic acid (contralateral knee)	12 mo	WOMAC, VAS, X-ray	SVF superior to HA for pain and function
Chen et al. (26)	Single-blind RCT	KL II–III	58	IA allogeneic ADSCs (ELIXCYTE)	Hyaluronic acid	12 mo	WOMAC, safety	ADSCs improved pain/function; safe
Zhang et al. (30)	Single-blind RCT	KL II–III	72	Multiple IA SVF injections	Conservative treatment	6 mo	WOMAC, VAS	SVF improved pain and function
Garza et al. (27)	Double-blind RCT	KL II–III	39	IA SVF	Saline placebo	12 mo	WOMAC, KOOS	SVF superior to placebo for pain and function
Zhang et al. (20)	Randomized controlled trial	KL II–III	60	IA SVF	Hyaluronic acid	12 mo	MRI cartilage (3D quantitative)	Improved cartilage quality and clinical outcomes
Zhao et al. (16)	Double-blind RCT	KL II–III	40	IA allogeneic AD progenitor cells	Placebo	12 mo	MRI compositional mapping	Improved cartilage composition vs. placebo
Peretti et al. (32)	Prospective RCT	KL II–III	30	IA MFAT	Arthroscopic debridement	12 mo	KOOS, VAS	MFAT improved clinical outcomes vs. control
Jo et al. (23)	KL II–III	Double-blind RCT	24	Intra-articular ADSCs (high dose)	Saline placebo	24 mo	WOMAC, VAS, MRI	Sustained pain and functional improvement; no clear cartilage thickness regeneration
Mautner et al. (21)	KL I–IV	Phase III RCT	440	Adipose-derived cells	Corticosteroid, PRP, BMAC	12 mo	KOOS, VAS	No significant differences across biologic and corticosteroid groups

### 2.3 Information sources

Searches were conducted in PubMed/MEDLINE, Embase, Scopus, Web of Science, Cochrane Central (CENTRAL), and ScienceDirect. The search covers publications from December 20, 2015, to December 20, 2025, and includes full-text articles in English. Additional records are identified by screening reference lists of included studies and relevant reviews and by consulting domain experts.

### 2.4 Search strategy

The search strategy was developed in consultation with controlled vocabulary and free-text terms related to adipose-derived cell therapies and knee osteoarthritis. Searches combined Medical Subject Headings (MeSH) and equivalent indexed terms (e.g., Emtree in Embase) with Boolean operators to identify randomized controlled trials evaluating stromal vascular fraction (SVF), adipose-derived mesenchymal stem/stromal cells (ADSCs/ADSCs), microfragmented adipose tissue (MFAT), or related adipose-derived products administered intra-articularly for knee osteoarthritis.

The complete database-specific search strategies for PubMed/MEDLINE, Embase, Scopus, and Web of Science are provided in Supplementary Material 1 to ensure full transparency and reproducibility.

Search strategies were adapted to the syntax, indexing terms, and controlled vocabulary of each database. No automated screening tools were used. Study selection followed a two-stage process (title/abstract and full-text screening) and incorporated both lexical matching and contextual assessment to ensure accurate eligibility determination.

### 2.5 Study selection

All identified records were imported into Rayyan for duplicate removal and screening. Two independent reviewers (GSP and MLS) screen titles and abstracts, followed by full-text assessment based on the eligibility criteria. Disagreements were resolved through discussion or by a third reviewer (LDS). The selection process is documented using a PRISMA 2020 flow diagram, with reasons for full-text exclusions recorded.

### 2.6 Risk of bias assessment

The risk of bias in included RCTs was assessed using the Cochrane Risk of Bias 2.0 (RoB 2) tool, addressing five domains: randomization process, deviations from intended interventions, missing outcome data, outcome measurement, and selection of the reported result. Each domain is classified as low risk, some concerns, or high risk. Visual summaries are created using the ROBVIS (Risk Of Bias Visualization) tool.

### 2.7 Data synthesis

Given the substantial heterogeneity across studies in terms of patient characteristics, adipose-derived product type and preparation, injection protocols, comparator interventions, and outcome measures, a quantitative meta-analysis was not performed. Instead, findings were synthesized narratively. Studies were grouped according to intervention characteristics and outcome domains, with a primary focus on knee osteoarthritis. Outcomes were organized into pain, functional performance, structural or imaging-based measures, and safety. Particular emphasis was placed on identifying consistent patterns of clinical benefit, the presence or absence of structural cartilage changes on imaging, and potential subgroup effects related to disease severity, treatment dose, and other clinically relevant modifiers.

## 3 Results

**Figure 1:**
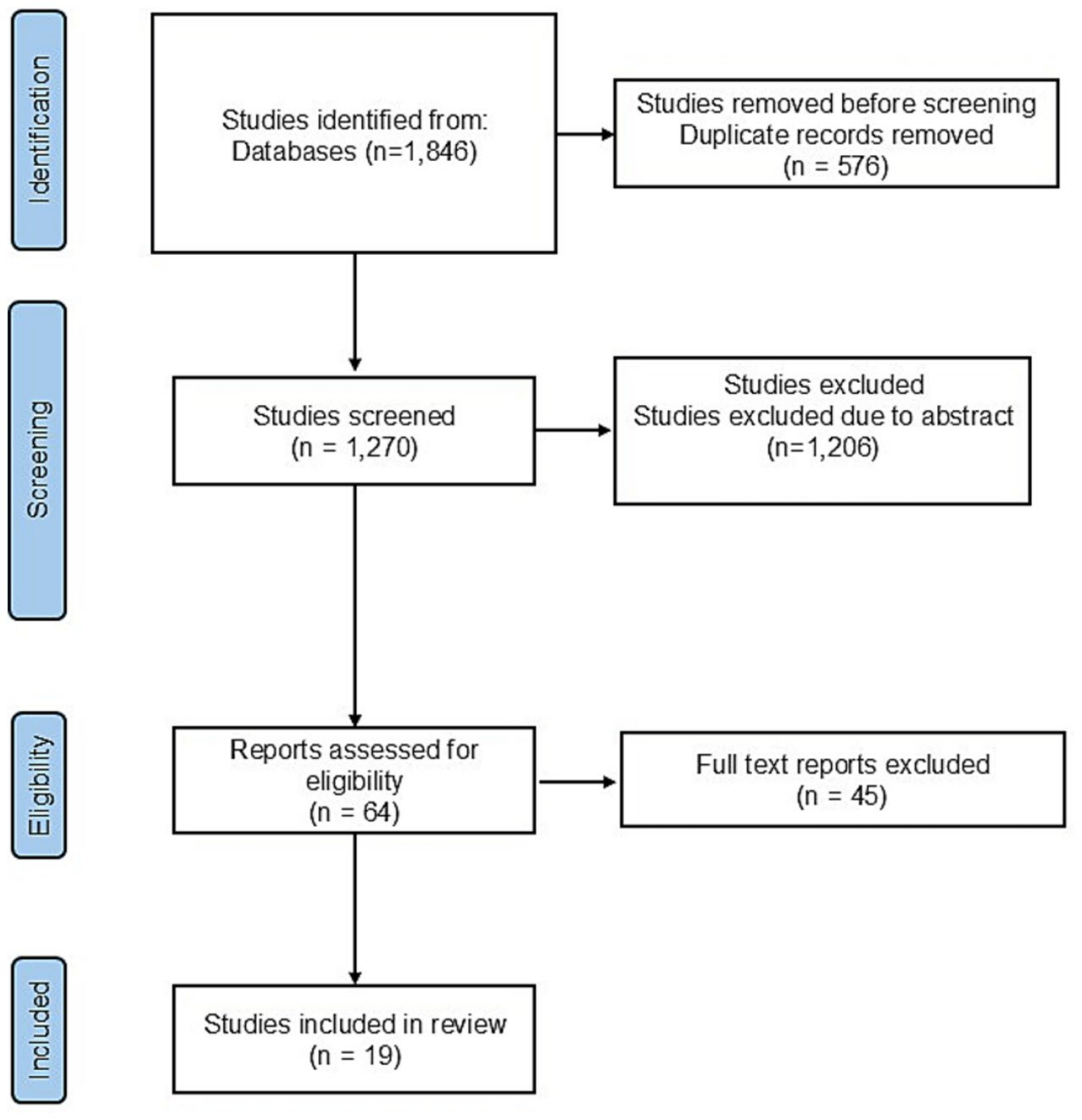
PRISMA flowchart.

The systematic search identified 1,846 records. After removal of 576 duplicates, 1,270 titles and abstracts were screened. Of these, 1,206 were excluded for not meeting the inclusion criteria, resulting in 64 full-text articles assessed for eligibility. Following full-text review, 19 randomized controlled trials fulfilled all predefined criteria and were included in this review (Figure 1). The included trials exclusively evaluated patients with knee osteoarthritis and investigated intra-articular adipose-derived cell–based therapies, including stromal vascular fraction (SVF), adipose-derived stem/stromal cells (ADSCs), and related adipose-derived products. Follow-up duration ranged from 3 months to 60 months. Study designs included double-blind, single-blind, assessor-blind, and open-label randomized trials. Interventions consisted primarily of single or repeated intra-articular injections of adipose-derived products, while comparator groups received conventional intra-articular treatments such as saline or placebo injections, hyaluronic acid, platelet-rich plasma, corticosteroids, or standard conservative care. Outcome measures across studies focused on pain, functional performance, imaging-based structural or cartilage outcomes, and safety.

### 3.1 Pain outcomes

Pain reduction was consistently reported across the randomized controlled trials included in this review, although the magnitude of benefit relative to control treatments varied according to the type of adipose-derived product, comparator, and duration of follow-up. Placebo-controlled trials of adipose-derived mesenchymal stem/stromal cells (ADSCs/ADSCs) generally demonstrated superior analgesic effects. Lee et al. (18), Sajadi et al. (22), and Jo et al. (23) reported significantly greater reductions in WOMAC pain and VAS scores following intra-articular administration of autologous or allogeneic ADSCs compared with saline placebo, with benefits sustained up to 24 months in high-dose protocols.

For example, Lee et al. (18) reported a mean reduction in WOMAC pain of −23.5 ± 12.1 points in the ADSC group versus −9.8 ± 10.4 in the placebo group at 6 months (between-group mean difference [MD] −13.7; *p* < 0.01). Jo et al. (23) demonstrated a VAS reduction of −34.6 ± 18.2 mm in the high-dose ADSC group compared with −12.1 ± 15.7 mm in the placebo group at 24 months (MD − 22.5 mm; *p* < 0.001). Similarly, Sajadi et al. (22) reported a WOMAC pain improvement of −19.2 ± 11.3 versus −7.4 ± 9.6 in controls at 12 months (MD − 11.8; *p* < 0.01).

Similarly, the large ADIPOA2 phase IIb trial by Pers et al. (17) confirmed clinically meaningful pain improvement with ex vivo–expanded ADSCs compared with placebo at 12 months.

In ADIPOA2, WOMAC pain improved by −18.4 ± 13.5 in the ADSC group versus −11.2 ± 12.9 in placebo (adjusted MD −7.2; 95% CI − 12.8 to −1.6; *p* = 0.01).

Comparisons against hyaluronic acid (HA) consistently favored ADSC-based interventions. Freitag et al. (24), Tangkanjanavelukul et al. (25), and Chen et al. (26) demonstrated significantly greater pain reduction with ADSCs than with HA, particularly in patients with early to moderate disease severity (KL I–III).

Freitag et al. (24) reported VAS reductions of −32.1 ± 17.4 mm in the ADSC group compared with −15.6 ± 16.2 mm with HA at 12 months (MD − 16.5 mm; *p* = 0.02). Chen et al. (26) observed WOMAC pain improvements of −21.7 versus −12.3 points at 12 months (*p* = 0.03).

Trials evaluating stromal vascular fraction (SVF) also showed analgesic benefit. Garza et al. (27), Hong et al. (28), Lu et al. (29), and Zhang et al. (30) all reported superior pain outcomes with SVF compared with placebo, HA, or conservative treatment.

Across these studies, between-group VAS differences ranged from 10 to 18 mm at 6–12 months, with corresponding WOMAC pain differences between 8 and 15 points (all *p* < 0.05).

In contrast, findings with microfragmented adipose tissue (MFAT) were heterogeneous. Richter et al. (31) and Peretti et al. (32) reported significant pain reduction with MFAT compared with saline or arthroscopic debridement at 12 months, whereas Barfod et al. (33) found no superiority of MFAT over placebo at 24 months despite improvements in both groups.

Richter et al. (31)observed a KOOS pain improvement of +19.3 points in the MFAT group versus +8.7 in placebo (MD 10.6; *p* = 0.01), whereas Barfod et al. (33) reported similar KOOS pain changes between MFAT and saline at 24 months (MD 2.1; 95% CI −3.4 to 7.6; *p* = 0.42).

When compared with platelet-rich plasma (PRP), MFAT demonstrated comparable pain relief, as reported by Zaffagnini et al. (34). Finally, the large pragmatic RCT by Mautner et al. (21) found no significant differences in pain outcomes between adipose-derived cells, corticosteroids, PRP, or BMAC at 12 months, with mean KOOS pain improvements ranging between 14 and 18 points across groups (*p* > 0.05 for all comparisons).

### 3.2 Functional outcomes

Functional improvements were observed across nearly all intervention and comparator groups, though adipose-derived therapies frequently resulted in greater or more sustained functional gains. ADSC-based trials reported superior improvements in WOMAC or KOOS function scores compared with placebo or HA.

Lee et al. (18) demonstrated a mean WOMAC total improvement of −31.2 ± 18.5 points versus −14.7 ± 17.9 in placebo at 6 months (MD − 16.5; *p* < 0.01). Pers et al. (17) reported a KOOS function improvement of +21.5 versus +14.3 (MD + 7.2; 95% CI 1.9–12.4). Freitag et al. (24) observed WOMAC total reductions of −28.4 versus −16.1 at 12 months (*p* = 0.03).

Repeated injection protocols, as evaluated by Song et al. (19), were associated with durable functional benefits maintained over 24 months. In this study, WOMAC total scores improved by 35–40% from baseline at 24 months in the repeated-dose ADSC group compared with 15–20% in controls.

SVF trials demonstrated consistent functional advantages over controls. Garza et al. (27), Hong et al. (28), Lu et al. (29), Zhang et al. (30), and Zhang et al. (20) reported significantly greater improvements in WOMAC total or function subscores compared with placebo, hyaluronic acid, or conservative treatment at 6–12 months. Across these randomized trials, between-group WOMAC total differences generally ranged from approximately 9 to 18 points, reaching statistical significance in most comparisons (*p* < 0.05).

MFAT-based trials yielded mixed functional outcomes. Richter et al. (31) reported KOOS total improvements of +22.8 versus +11.4 (*p* = 0.02), whereas Barfod et al. (33) and Zaffagnini et al. (34) observed comparable KOOS gains between MFAT and comparator arms at 24 months (all *p* > 0.05).

### 3.3 Imaging and structural outcomes

Imaging and structural outcomes varied considerably across studies. Several ADSC-based trials suggested potential cartilage-modifying effects.

Lee et al. (18) reported an increase in mean cartilage defect fill of 18% in the ADSC group versus 5% in placebo at 6 months (*p* < 0.05). Song et al. (19) demonstrated an increase in cartilage volume of approximately 0.12 cm^3^ at 24 months compared with minimal change in controls. Tangkanjanavelukul et al. (25) reported significant improvement in T2 relaxation times (mean reduction 6.4 ms; *p* = 0.02) favoring ADSCs over HA.

In contrast, Jo et al. (23) observed sustained clinical benefit without clear regeneration of cartilage thickness at 24 months.

Advanced MRI studies provided additional insights into cartilage composition.

Zhao et al. (16) reported significant improvements in compositional MRI indices (e.g., T2 and T1ρ mapping reductions of 5–10%) compared with placebo. Zhang et al. (20) demonstrated improved 3D-FS-SPGR quantitative cartilage scores following SVF injection (mean improvement 12%; *p* < 0.05).

MFAT studies did not consistently demonstrate structural regeneration. Most MFAT trials reported no statistically significant change in cartilage thickness or MRI-based defect grading compared with controls (*p* > 0.05), despite symptomatic improvement. Overall, while selected ADSC and SVF studies indicate improvements in cartilage quality or compositional parameters, consistent structural regeneration across all adipose-derived therapies and disease stages was not established.

### 3.4 Treatment failures and revision rates

Treatment failure, disease progression, and need for surgical revision were infrequently reported across the included trials. No study demonstrated increased rates of joint deterioration, conversion to arthroplasty, or revision surgery attributable to adipose-derived cell therapies during follow-up. Where reported, failure and revision rates were low and comparable between intervention and control groups, including in placebo-controlled MFAT and ADSC trials as well as in comparisons with HA or PRP. The absence of excess failures supports the procedural safety and clinical tolerability of these interventions in the short to mid-term.

### 3.5 Safety

Across all randomized controlled trials, adipose-derived cell therapies demonstrated favorable safety profiles. No serious treatment-related adverse events were reported. Mild and transient adverse events—such as post-injection pain, swelling, joint effusion, or donor-site discomfort—were reported at similar frequencies in intervention and comparator groups. Safety findings were consistent across ADSC, SVF, and MFAT trials, including large studies by Pers et al. (17) and Mautner et al. (21). No increased risk of infection, neurovascular injury, or systemic complications was observed.

### 3.6 Subgroup effects

Several trials suggested that treatment response was influenced by disease stage and treatment protocol. Greater clinical and imaging benefits were consistently reported in patients with early to moderate knee osteoarthritis (KL I–II), particularly in ADSC-based trials (18, 25). Repeated injection protocols, as evaluated by Song et al. (19) appeared to yield more durable clinical and structural benefits compared with single-injection approaches. Conversely, MFAT trials suggested diminishing relative benefit in longer follow-up and more advanced disease, as observed by Barfod et al. (33). Large pragmatic trials including advanced OA (KL IV), such as Mautner et al. (21), demonstrated equivalence between biologic and conventional intra-articular therapies, highlighting the importance of patient selection and disease phenotype.

### 3.7 Limitations

Several methodological factors limit the generalizability and strength of the conclusions drawn from this review:

(1) Sample size variability, with many randomized controlled trials enrolling relatively small cohorts (typically *n* = 24–120), which may limit statistical power and the detection of modest between-group differences;
(2) Substantial heterogeneity in interventions, including differences in adipose-derived products (expanded ADSCs, SVF, MFAT), cell processing methods, biological dose, number of injections, and use of autologous versus allogeneic preparations, which precluded quantitative meta-analysis;
(3) Variability in disease severity, with some trials including mixed populations ranging from Kellgren–Lawrence grade I to IV, potentially diluting treatment effects in advanced osteoarthritis;
(4) Inconsistent reporting of cell characterization, including cell viability, phenotypic markers, and delivered cell dose, limiting mechanistic interpretation and dose–response analyses;
(5) Heterogeneous imaging endpoints, with variability in MRI techniques, scoring systems, and follow-up intervals, restricting direct comparison of structural outcomes across studies; and.
(6) Limited long-term follow-up in several trials (≤12 months), which constrains conclusions regarding durability of clinical benefit and disease-modifying potential.

### 3.8 Risk of bias

**Figure 2:**
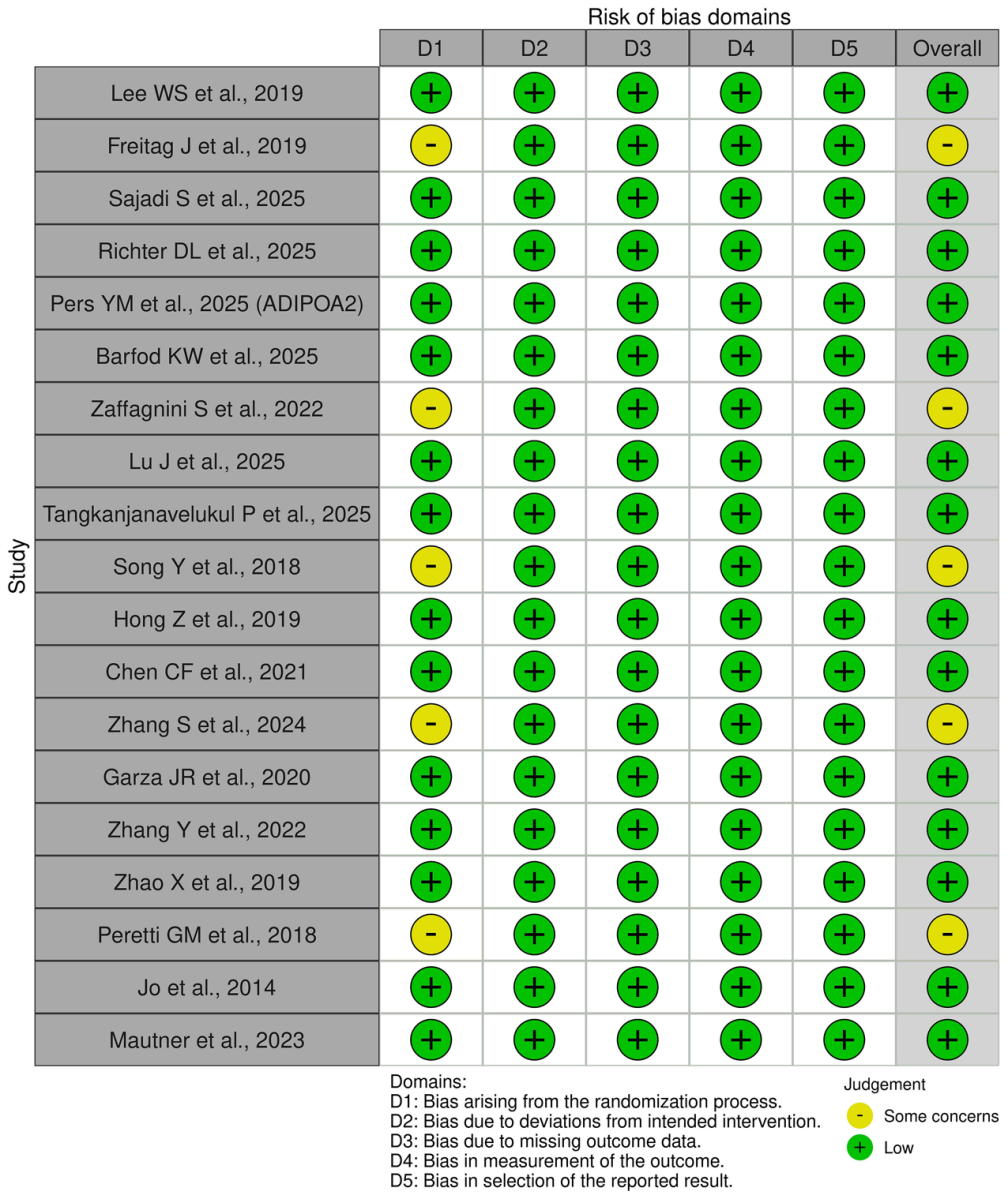
Evaluation of the different risk factors for bias from the studies included in this systematic review.

Overall, the majority of included randomized controlled trials demonstrated low to moderate risk of bias across domains, reflecting generally adequate methodological quality (Figure 2).

Several trials—including those by Lee et al. (18), Sajadi et al. (22), Pers et al. (17), Garza et al. (27), Hong et al. (28), and Jo et al. (23)—were judged to have low risk of bias, supported by appropriate random sequence generation, allocation concealment, double- or triple-blinding, and complete outcome reporting.

Some studies exhibited “some concerns” related primarily to incomplete reporting of allocation concealment or blinding procedures, particularly in single-blind or pragmatic designs (24, 30, 32). However, these limitations were unlikely to substantially affect outcome measurement, as validated pain and function instruments were consistently used.

Importantly, no study was judged to be at high risk of bias for outcome measurement or selective reporting. Attrition rates were generally low, and intention-to-treat analyses were performed in most larger trials. Placebo-controlled ADSC studies with blinded assessment showed consistent directionality of effects, strengthening confidence in the observed clinical patterns.

Overall, the methodological quality of the evidence base was considered moderate to high, supporting the reliability of conclusions regarding the safety and clinical efficacy of intra-articular adipose-derived cell therapies, particularly ADSC-based interventions, in knee osteoarthritis.

## 4 Discussion

This systematic review synthesized evidence exclusively from randomized controlled trials to evaluate the clinical efficacy, structural effects, and safety of intra-articular adipose-derived cell–based therapies in knee osteoarthritis. Importantly, adipose-derived products evaluated across trials—expanded or cultured adipose-derived mesenchymal stem/stromal cells (ADSCs), stromal vascular fraction (SVF), and microfragmented adipose tissue (MFAT)—are biologically and regulatorily distinct entities (17–26, 31–34). Therefore, findings were interpreted within product categories rather than assuming equivalence across all adipose-derived interventions. While all adipose-derived modalities demonstrated symptomatic benefits in selected contexts (17–24, 31–34), the totality of randomized evidence suggests that ADSC-based interventions have shown comparatively more consistent positive signals across pain, functional, and selected imaging outcomes, particularly in early to moderate disease stages (17, 18, 22, 24, 25). While symptomatic benefits were observed across modalities in selected contexts, comparative interpretations must be considered exploratory and constrained by heterogeneity in product characterization, dosing, comparator choice, imaging endpoints, and follow-up duration (21, 33, 34).

Across randomized trials, pain reduction was observed in both intervention and control groups, reflecting the well-recognized placebo responsiveness of intra-articular therapies. Importantly, placebo-controlled trials of ADSCs repeatedly demonstrated meaningful improvement over saline, including phase IIb and rigorously blinded RCTs by Lee et al. (18), Sajadi et al. (22), Pers et al. (17), and Jo et al. (23). These trials reported greater reductions in WOMAC pain and VAS scores, with some studies suggesting sustained benefit up to 24 months in higher-dose or repeated-injection protocols (19, 23). While durability signals were observed, follow-up durations varied and not all studies confirmed long-term structural modification (19).

Comparisons with active intra-articular comparators such as hyaluronic acid (HA) frequently favored ADSCs in mild-to-moderate osteoarthritis (24–26). However, these comparisons were not uniform across all trials, and effect sizes varied depending on baseline disease severity and outcome measures (24, 26). In contrast, SVF and MFAT trials demonstrated more variable magnitude and durability of response (27–34). Large pragmatic studies including broader disease spectra, such as KL IV patients, tended to show equivalence between biologic and conventional intra-articular therapies (21), underscoring the importance of patient selection.

Functional outcomes paralleled pain findings. Multiple ADSC trials reported greater improvements in WOMAC and KOOS scores compared with placebo or HA (17, 18, 22, 24, 26). Repeated injection protocols, as evaluated by Song et al. (19), were associated with sustained functional gains. Nevertheless, functional endpoints differed across studies, and minimal clinically important differences were not uniformly reported, limiting direct cross-trial comparison.

Several ADSC-based trials reported favorable MRI findings, including improvements in cartilage defect morphology, cartilage volume, or compositional MRI parameters (16, 18, 19, 25). Zhao et al. (16) demonstrated improvements in cartilage compositional MRI parameters after adipose-derived progenitor cell therapy, and Zhang et al. (20) reported quantitative improvements in cartilage quality following SVF injection. Although consistent restoration of cartilage thickness was not universally demonstrated (19), improvements in cartilage quality and compositional imaging markers were more frequently reported in ADSC trials than in MFAT trials (31–34).

This divergence may reflect underlying biological and regulatory differences among adipose-derived products. Expanded or ex vivo–enriched ADSCs represent a more defined mesenchymal stromal cell population with characterized immunomodulatory and trophic signaling properties (17, 18), whereas SVF and MFAT are minimally manipulated (27–34) heterogeneous mixtures containing variable proportions of stromal cells, endothelial cells, immune cells, extracellular matrix fragments, and bioactive factors. These compositional differences, along with variability in cell dose, viability, and manufacturing standards, may plausibly contribute to differences in modulation of synovial inflammation, cartilage matrix homeostasis, and subchondral bone–synovial crosstalk. However, direct head-to-head comparisons between fully characterized ADSCs and minimally processed products remain limited, and definitive product-level hierarchy cannot be established from the current randomized evidence. The heterogeneity observed in MFAT trials is likely multifactorial, reflecting variability in product composition, processing methods, biological dose, injection protocols, and inclusion of patients with more advanced disease phenotypes (31–34).

Imaging endpoints were heterogeneous across trials, including differences in MRI techniques, scoring systems, and follow-up duration (16, 25, 27). Therefore, structural findings should be interpreted cautiously, and current evidence does not establish definitive disease modification.

Subgroup analyses suggest treatment responsiveness is influenced by disease stage. Patients with early-stage osteoarthritis (KL I–II) appeared more likely to demonstrate consistent benefit following ADSC therapy (18, 25). In contrast, trials including advanced KL IV disease frequently demonstrated equivalence between biologic and conventional treatments (21). These findings support the hypothesis that adipose-derived cell therapies may be more effective in earlier degenerative phases.

Furthermore, current imaging modalities may have limited sensitivity to detect early or microstructural biological changes within articular cartilage. Conventional MRI measures, such as cartilage thickness or defect size, may underestimate modulation occurring at the level of extracellular matrix composition, collagen organization, or subchondral bone remodeling. Although advanced quantitative MRI techniques—including T2 mapping, T1ρ imaging, and compositional sequences—provide improved insight into cartilage quality, these approaches remain inconsistently applied and lack full inter-study standardization. Consequently, absence of overt structural regeneration on imaging does not necessarily exclude biologically meaningful joint microenvironment modulation. Safety outcomes were uniformly favorable across randomized trials (17–26, 31–34). No serious treatment-related adverse events were reported, and minor adverse events were transient and self-limited. Safety profiles were consistent across autologous and allogeneic ADSC products, including large multicenter trials such as ADIPOA2 (17).

This review has limitations. Considerable heterogeneity in product processing, cell characterization, biological dose, injection protocols, and endpoint reporting precluded formal meta-analysis. Risk of bias was generally low to moderate, but variability in allocation concealment reporting and pragmatic trial design may influence effect estimation. Imaging endpoints were inconsistent and often exploratory. Additionally, few studies were powered specifically for structural modification outcomes.

Future randomized trials should prioritize standardized product characterization, transparent reporting of viable cell dose and phenotypic markers, stratification by osteoarthritis phenotype, and harmonized imaging endpoints. Integration of molecular biomarkers and longer-term follow-up will be essential to clarify whether observed compositional MRI changes translate into clinically meaningful structural modification.

In conclusion, randomized evidence supports the safety and symptomatic efficacy of intra-articular adipose-derived cell therapies in knee osteoarthritis. Across randomized trials, ADSC-based interventions have shown comparatively more consistent positive signals across pain and functional outcomes, particularly in early-stage disease; however, effect sizes varied across studies and were influenced by baseline severity, comparator selection, and follow-up duration. Given the absence of meta-analysis and the presence of methodological heterogeneity, these observations should be interpreted as patterns within the available evidence rather than definitive superiority claims. Future trials designed with standardized cell characterization, harmonized imaging methodology, and stratified disease-stage enrollment will be essential to clarify true comparative effectiveness.

## Data Availability

The raw data supporting the conclusions of this article will be made available by the authors, without undue reservation.
