# Supplementary material for: Intra-articular adipose-derived cell therapies for knee osteoarthritis: a systematic review of randomized controlled trials
Source: Front Med (Lausanne). 2026 Apr 22;13:1771008. doi: 10.3389/fmed.2026.1771008 (PMC13147480; doi:10.3389/fmed.2026.1771008)
Supplement: Supplementary file 1 [file Table_1.docx]

**Supplementary Material 1**

**Complete Database-Specific Search Strategies**

The following search strategies were used to identify randomized controlled trials evaluating intra-articular adipose-derived cell–based therapies for knee osteoarthritis. Searches were conducted in PubMed/MEDLINE, Embase, Scopus, and Web of Science. Searches were limited to human studies published in English between January 1, 2015 and December 31, 2025.

**1. PubMed/MEDLINE**

("Osteoarthritis, Knee"[Mesh] OR "knee osteoarthritis" OR "gonarthrosis")
AND
("Adipose-Derived Stem Cells"[Mesh] OR "adipose-derived stem cells" OR "adipose-derived stromal cells" OR "adipose-derived mesenchymal stem cells" OR "ADSC" OR "AD-MSC" OR "stromal vascular fraction" OR "SVF" OR "microfragmented adipose tissue" OR "MFAT")
AND
("Injections, Intra-Articular"[Mesh] OR "intra-articular injection")
AND
("Randomized Controlled Trial"[Publication Type] OR "randomized controlled trial" OR "randomised controlled trial" OR "RCT")

**2. Embase (Emtree)**

('knee osteoarthritis'/exp OR 'knee osteoarthritis' OR gonarthrosis)
AND
('adipose derived stem cell'/exp OR 'adipose-derived stem cells' OR 'adipose-derived stromal cells' OR 'adipose-derived mesenchymal stem cells' OR ADSC OR 'stromal vascular fraction' OR SVF OR 'microfragmented adipose tissue' OR MFAT)
AND
('intraarticular drug administration'/exp OR 'intra-articular injection')
AND
('randomized controlled trial'/exp OR 'randomized controlled trial' OR RCT)

**3. Scopus**

TITLE-ABS-KEY("knee osteoarthritis" OR gonarthrosis)
AND TITLE-ABS-KEY("adipose-derived stem cells" OR "adipose-derived stromal cells" OR "adipose-derived mesenchymal stem cells" OR ADSC OR "stromal vascular fraction" OR SVF OR "microfragmented adipose tissue" OR MFAT)
AND TITLE-ABS-KEY("intra-articular injection")
AND TITLE-ABS-KEY("randomized controlled trial" OR RCT)

**4. Web of Science**

TS=("knee osteoarthritis" OR gonarthrosis)
AND TS=("adipose-derived stem cells" OR "adipose-derived stromal cells" OR "adipose-derived mesenchymal stem cells" OR ADSC OR "stromal vascular fraction" OR SVF OR "microfragmented adipose tissue" OR MFAT)
AND TS=("intra-articular injection")
AND TS=("randomized controlled trial" OR RCT)
